# ppiPre: predicting protein-protein interactions by combining heterogeneous features

**DOI:** 10.1186/1752-0509-7-S2-S8

**Published:** 2013-10-14

**Authors:** Yue Deng, Lin Gao, Bingbo Wang

**Affiliations:** 1 School of Computer Science and Technology, Xidian University, Xi'an 710071, PR China; 2 Institute of Software Engineering, Xidian University, Xi'an 710071, PR China

## Abstract

**Background:**

Protein-protein interactions (PPIs) are crucial in cellular processes. Since the current biological experimental techniques are time-consuming and expensive, and the results suffer from the problems of incompleteness and noise, developing computational methods and software tools to predict PPIs is necessary. Although several approaches have been proposed, the species supported are often limited and additional data like homologous interactions in other species, protein sequence and protein expression are often required. And predictive abilities of different features for different kinds of PPI data have not been studied.

**Results:**

In this paper, we propose ppiPre, an open-source framework for PPI analysis and prediction using a combination of heterogeneous features including three GO-based semantic similarities, one KEGG-based co-pathway similarity and three topology-based similarities. It supports up to twenty species. Only the original PPI data and gold-standard PPI data are required from users. The experiments on binary and co-complex gold-standard yeast PPI data sets show that there exist big differences among the predictive abilities of different features on different kinds of PPI data sets. And the prediction performance on the two data sets shows that ppiPre is capable of handling PPI data in different kinds and sizes. ppiPre is implemented in the R language and is freely available on the CRAN (http://cran.r-project.org/web/packages/ppiPre/).

**Conclusions:**

We applied our framework to both binary and co-complex gold-standard PPI data sets. The detailed analysis on three GO aspects suggests that different GO aspects should be used on different kinds of data sets, and that combining all the three aspects of GO often gets the best result. The analysis also shows that using only features based solely on the topology of the PPI network can get a very good result when predicting the co-complex PPI data. ppiPre provides useful functions for analysing PPI data and can be used to predict PPIs for multiple species.

## Background

Although different experimental methods [1,2] have already generated a large amount of PPI for many model species in recent years [3], these existing PPI data are incomplete and contain many false positive interactions. In order to refine these PPI data, computational approaches are urgently needed.

Some recent researches have shown that PPIs can be integrated with other kinds of biological data in using supervised learning to predict PPIs [4-7]. In supervised learning, a classifier is trained using truly interacting protein pairs (positive samples) and protein pairs which are not interacting with each other (negative samples). Then the trained classifier is able to recover false negative interactions and remove false positive interactions from the PPIs input by users.

Existing studies are mainly differing in the selection of features used in the prediction framework. In these studies, different biological evidences are extracted and used as features training the classifier, including Gene Ontology (GO) functional annotations [8,9], protein sequences [10] and co-expressed proteins [11]. For the organisms or proteins which are lack of research, biological features may don't work well, so features based on network topology are also needed to integrate [12-14].

Although some frameworks and tools have also been proposed for predicting PPIs [15-20], they have two disadvantages in general. First, most of the frameworks only support a few well studied model organisms. Second, these frameworks often need users to provide additional biological data along with the PPIs. Moreover, different species often require different features, which make these existing frameworks not very convenient to use.

In this paper, we describe ppiPre, an open-source framework for the PPI prediction problem. The framework is implemented in the R language so it can work together with other R packages dealing with biological data and network [21], which is different from other tools accessed via web services. ppiPre integrates features extracted from multiple heterogeneous data sources, including GO [22], KEGG [23] and topology of the PPI network. Users don't need to provide additional biological data other than gold-standard PPI data. ppiPre provides functions for measuring the similarity between proteins and for predicting PPIs from the existing PPI data.

## Methods

Heterogeneous features are integrated in the prediction framework of ppiPre, including three GO-based semantic similarities, one KEGG-based similarity indicating the proteins are involved in the same pathways and three topology-based similarities using only the network structure of the PPI network.

We chose these three features to be integrated in our framework because they are highly available for the PPIs of different species and can be easily accessed in the R environment. Not like other methods and software tools, ppiPre did not integrate biological features that may not be available for the species or proteins which are not well studied, such as structural and domain information.

### GO-based semantic similarities

Proteins are annotated by GO with terms from three aspects: biological process (BP), molecular function (MF), and cellular component (CC). Directed acyclic graphs (DAGs) are used to describe these aspects. It is known that interacting protein pairs are likely to be involved in similar biological processes or in similar cellular component compared to those non-interacting proteins [2,24,25]. Thus if two proteins are semantically similar based on GO annotation, the probability that they actually interact is higher than two proteins that are less similar.

Several similarity measures have been developed for evaluating the semantic similarity between two GO terms [26-28]. The information content (IC) of GO terms and the structure of the GO DAG are often used in these measures.

The IC of a term *t *can be defined as follows:

(1) $$IC\left(t\right)\text{ = - }\text{log}\left(p\left(t\right)\right)$$

where p(t) is the probability of occurrence of the term *t *in a certain GO aspect. Two IC-based semantic similarity measures proposed recently are integrated in ppiPre, which are Topological Clustering Semantic Similarity (TCSS) [29] and IntelliGO [30].

#### TCSS

In TCSS, the GO DAGs are divided into subgraphs. A PPI is scored higher if the two proteins are in the same subgraph. The algorithm is made up of two major steps.

In the first step, a threshold on the ICs of all terms is used to generate multiple subgraphs. The roots of the subgraphs are the terms which are below the previously defined threshold. If roots of two subgraphs have similar IC values, these two subgraphs are merged. Overlapping subgraphs may occur because some GO terms have more than one parent terms. In order to remove overlap between subgraphs, edge removal and term duplication are processed. Transitive reduction of GO DAG is used to remove overlapping edges by generating the smallest graph that has the same transitive closure as the original subgraph. After edge removal, if a term is included in two or more subgraphs, it will be duplicated into each subgraph. More details are described in [29].

After the first step, a meta-graph is constructed by connecting all subgraphs. Then the second step called normalized scoring is processed. For two GO terms, normalized semantic similarity is calculated based on the meta-graph rather than the whole GO DAG so that more balanced semantic similarity scores can be obtained.

Using the frequency of proteins that are annotated to GO term *t *and its children, the information content of annotation (ICA) for a GO term *t *is:

(2) $$ICA\left(t\right)=\text{ - }\text{ln}\left(\frac{\left|P_{t}\underset{c\in N\left(t\right)}{⋃}P_{c}\right|}{\underset{t\in O}{\sum }\left|P_{t}\underset{c\in N\left(t\right)}{⋃}P_{c}\right|}\right)$$

where *P_t _*is the proteins that are annotated by *t *in aspect *O *and *N*(*t*) is the child terms of *t*.

The information content of subgraph (ICS) for term $t_{m}^{s}$ in the *m^th ^*subgraph $G_{m}^{s}$ is defined as follows:

(3) $$ICS\left(t_{m}^{s}\right)=\frac{ICA\left(t_{m}^{s}\right)}{\underset{t_{m}^{s}\in G_{m}^{s}}{\text{max}}ICA\left(t_{m}^{s}\right)}$$

The information content of meta-graph (ICM) for a term $t_{n}^{m}$ in meta-graph *G^m ^*is defined as follows:

(4) $$ICM\left(t_{n}^{m}\right)=\frac{ICA\left(t_{n}^{m}\right)}{\underset{t_{n}^{m}\in G^{m}}{\text{max}}ICA\left(t_{n}^{m}\right)}$$

Finally, the similarity between two proteins *i *and *j *is defined as:

(5) $$Sim_{TCSS}\left(i,j\right)=\underset{s_{m}\text{,}t_{n}\in T_{i}\text{,}T_{j}}{\text{max}}\left\{\begin{array}{c} ICM_{\text{max}}\left(LCA\left(s_{m}\text{,}t_{n}\right)\right)\;if\;s_{m}\in G_{m}^{s}\;and\;t_{n}\in G_{n}^{s}\\ ICS_{\text{max}}\left(LCA\left(s_{m}\text{,}t_{n}\right)\right)\;if\;s_{m}\text{,}t_{n}\in G_{n}^{s} \end{array}\right)$$

where *LCA*(*s_m_*,*t_n_*) is the common ancestor of the terms *s_m _*and *t_n _*with the highest IC. *T_i _*and *T_j _*are two sets of GO terms which annotate the two proteins *i *and *j *respectively.

#### IntelliGO

The IntelliGO similarity measure introduces a novel annotation vector space model. The coefficients of each GO term in the vector space consider complementary properties. The IC of a specific GO term and its evidence code (EC) [31] are used to assign this GO term to a protein. The coefficient *α_t _*given to term *t *is defined as follows:

(6) $$\alpha_{t}=w\left(g\text{, }t\right)*IAF\left(t\right)$$

where *w*(*g*, *t*) is the weight of the EC which indicates the annotation origin between protein *g *and GO term *t*, and IAF (Inverse Annotation Frequency) represents the frequency of term *t *occurred in all the proteins annotated in the aspect where *t *belongs.

For two proteins *i *and *j*, the IntelliGO uses their vectorial representation $\vec{i}$ and $\vec{j}$ to measure their similarity, which is defined as follows:

(7) $$Sim_{IntelliGO}\left(i\text{,}j\right)\text{ = }\frac{\vec{i}*\vec{j}}{\sqrt{\vec{i}*\vec{i}}*\sqrt{\vec{j}*\vec{j}}}$$

The detailed explanation of the definition can be found in [30].

#### Wang's method

The similarity measure proposed by Wang [32] is also implemented in the ppiPre package, which is based on the graph structure of GO DAG.

In the GO DAG, each edge has a type which is "is-a" or "part-of". In Wang's measure, a weight is given to each edge according to its type. *DAG_t _*= (*t*,*T_t_*,*E_t_*) represents the subgraph made up of term *t *and its ancestors, where *T_t _*is the set of the ancestor terms of *t *and *E_t _*is the set of edges in *DAG_t_*.

In *DAG_t_*, *S_t_*(*n*) measures the semantic contribution of term *n *to term *t*, which is defined as:

(8) $$\{\begin{array}{c} S_{t}\left(t\right)=1\\ S_{t}\left(n\right)=\max\left\{w_{e}{}^{*}S_{t}\left(n^{\prime }\right)|n^{\prime }\in childrenof\left(n\right)\right\}\text{if }t\neq n \end{array}$$

The similarity between two GO term *m *and term *n *is defined as:

(9) $$Sim_{Wang}\left(m\text{, }n\right)\text{ = }\frac{\underset{t\in T_{m}\cap T_{n}}{\sum }S_{m}\left(t\right)\text{ + }S_{n}\left(t\right)}{SV\left(m\right)\text{ + }SV\left(n\right)}$$

where *SV*(*m*) is the sum of the semantic contribution of all the terms in *DAG_m_*.

The semantic similarity between two proteins *i *and *j *is defined as the maximum value of all the similarity between any term that annotate *i *and any term that annotate *j*.

### KEGG-based similarity

Proteins that work together in the same KEGG pathway are likely to interact[33][34]. The KEGG-based similarity between proteins *i *and *j *is calculated using the co-pathway membership information in KEGG. The similarity is defined as:

(10) $$Sim_{KEGG}\left(i\text{,}j\right)=\frac{\left|P\left(i\right)\cap P\left(j\right)\right|}{\left|P\left(i\right)\cup P\left(j\right)\right|}$$

where *P*(*i*) is the set of pathways which protein *i *involved in the KEGG database.

### Topology-based similarities

In order to deal with the proteins that haven't got any annotations in GO or KEGG database, topology-based similarity measures are also integrated. In ppiPre, three different topological similarities are implemented.

The Jaccard similarity [35] between two proteins *i *and *j *is defined as:

(11) $$Sim_{Jac}\left(i\text{,}j\right)=\frac{\left|N\left(i\right)\cap N\left(j\right)\right|}{\left|N\left(i\right)\cup N\left(j\right)\right|}$$

where *N*(*i*) is set of all the direct neighbours of protein *i *in PPI network.

Adamic-Adar(AA) similarity [36] punishes the proteins with high degree by assigning more weights to the nodes with low degree in PPI network. The AA similarity between two proteins *i *and *j *is defined as:

(12) $$Sim_{AA}\left(i\text{,}j\right)=\underset{n\in N\left(i\right)\cap N\left(j\right)}{\sum }\frac{1}{\text{log}k_{n}}$$

where *k_n _*is the degree of protein *n*.

Resource Allocation (RA) similarity [37] is similar to AA similarity and considers the common neighbours of two nodes as resource transmitters. The RA similarity between two proteins *i *and *j *is defined as:

(13) $$Sim_{RA}\left(i\text{,}j\right)=\underset{n\in N\left(x\right)\cap N\left(y\right)}{\sum }\frac{1}{k_{n}}$$

### Prediction framework

The data of interacting protein pairs verified by experiments are very incomplete and the non-interacting protein pairs far outnumber interacting protein pairs. So the classical SVM [38] which is able to handle small and unbalanced data is chosen to integrate different features in ppiPre. We have tested different kernels in e1071 and the results showed no significant difference, so the default kernel and parameters are used in ppiPre.

**Figure 1:**
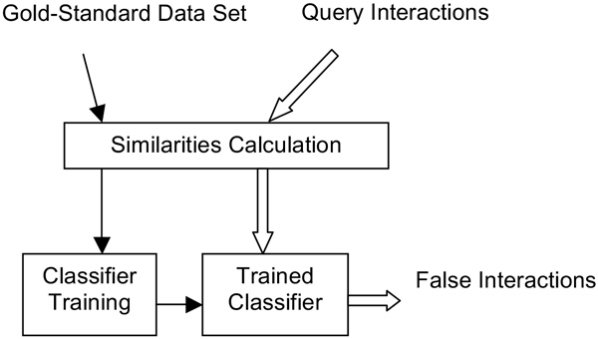
**Graphical overview of the prediction framework**. First, SVM is trained using the gold-standard PPI data sets (solid arrows). Then the trained classifier can be used to predict PPIs from the input PPI data (hollow arrows).

The prediction framework of ppiPre is presented in Figure 1. Heterogeneous features are calculated for the gold-standard PPI data set which is given by users, and the SVM classifier is trained by the gold-standard positive and negative data set (solid arrows). After the classifier is trained, the features are calculated from the query PPIs input by users, and the trained classifier can predict false positive and false negative PPIs from the input data (hollow arrows).

## Results and discussion

Since all the features are calculated within the package, users don't need to provide additional biological data for different species. When users use ppiPre to predict the PPIs, they only need to provide both the gold-standard positive and negative training set and the test set. In this paper, we test the performance of ppiPre in yeast using two yeast gold-standard positive data sets which are a high quality binary data set provided by Yu's research [39] and the MIPS data set [40]. Self-interactions and duplicate interactions were removed previously. The detail of the two gold-standard data sets is shown in Table 1.

**Table 1:** Gold-standard positive yeast protein interaction data sets

Data set	Number of Interactions	Number of Proteins	Interaction Type
Yu	1263	1078	binary
MIPS	8250	871	co-complex

Non-interacting pairs were randomly selected from the proteins in gold-standard positive data sets as gold-standard negative data sets. The positive and negative data sets are set to the same size. In order to minimize the impact to the topological characteristics of the PPI network, the degree of each protein was maintained.

10-fold cross validation was used to evaluate the performance of the prediction framework.

### Predictive abilities of GO-based similarities

**Figure 2:**
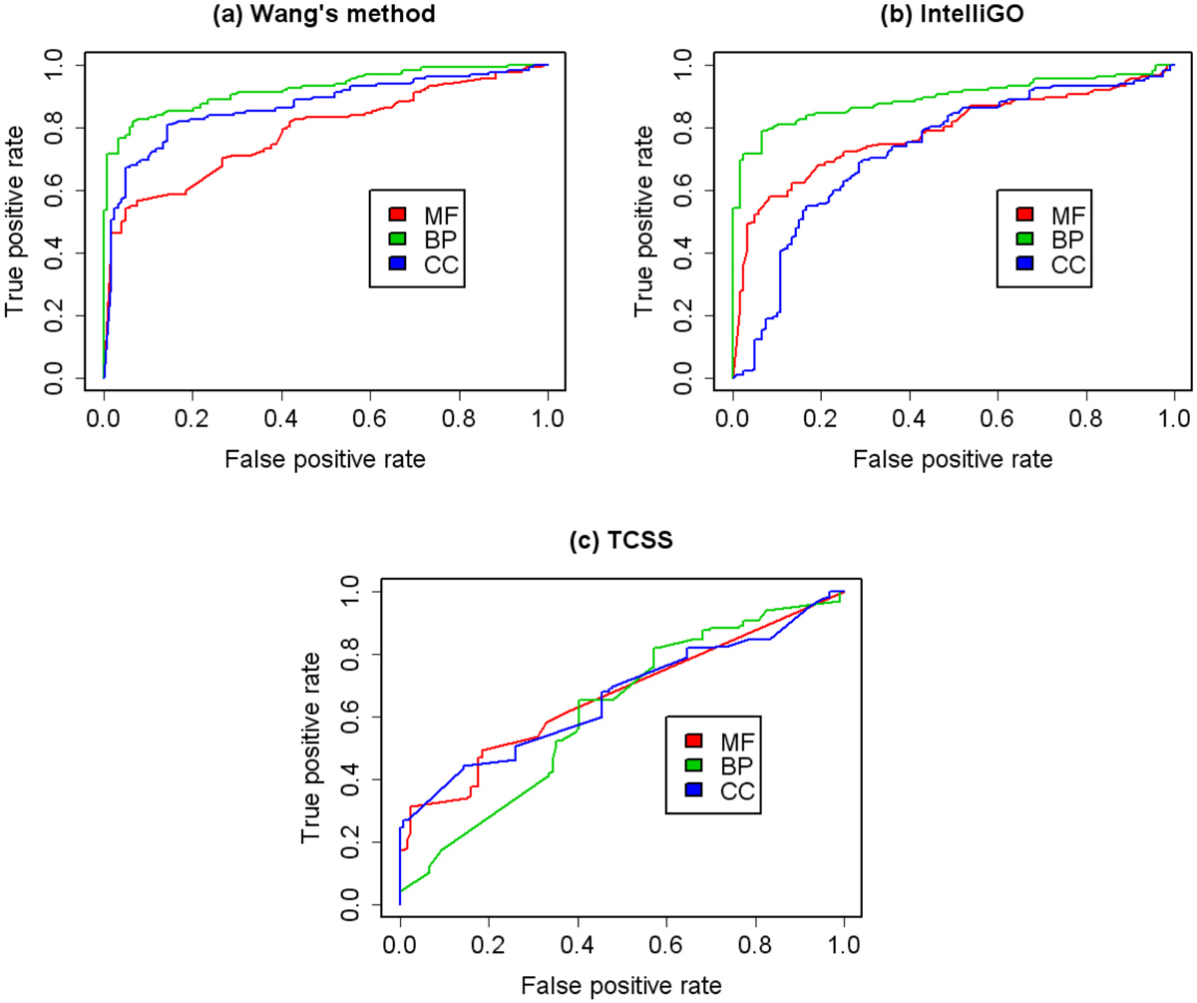
**ROC curves for binary data set using single GO aspect**. ROC evaluations of three GO aspects with three semantic similarity measures on the binary PPI data set are shown. The evaluation was performed using only one GO aspect at a time. BP shows the overall best predictive abilities in three aspects in GO.

**Figure 3:**
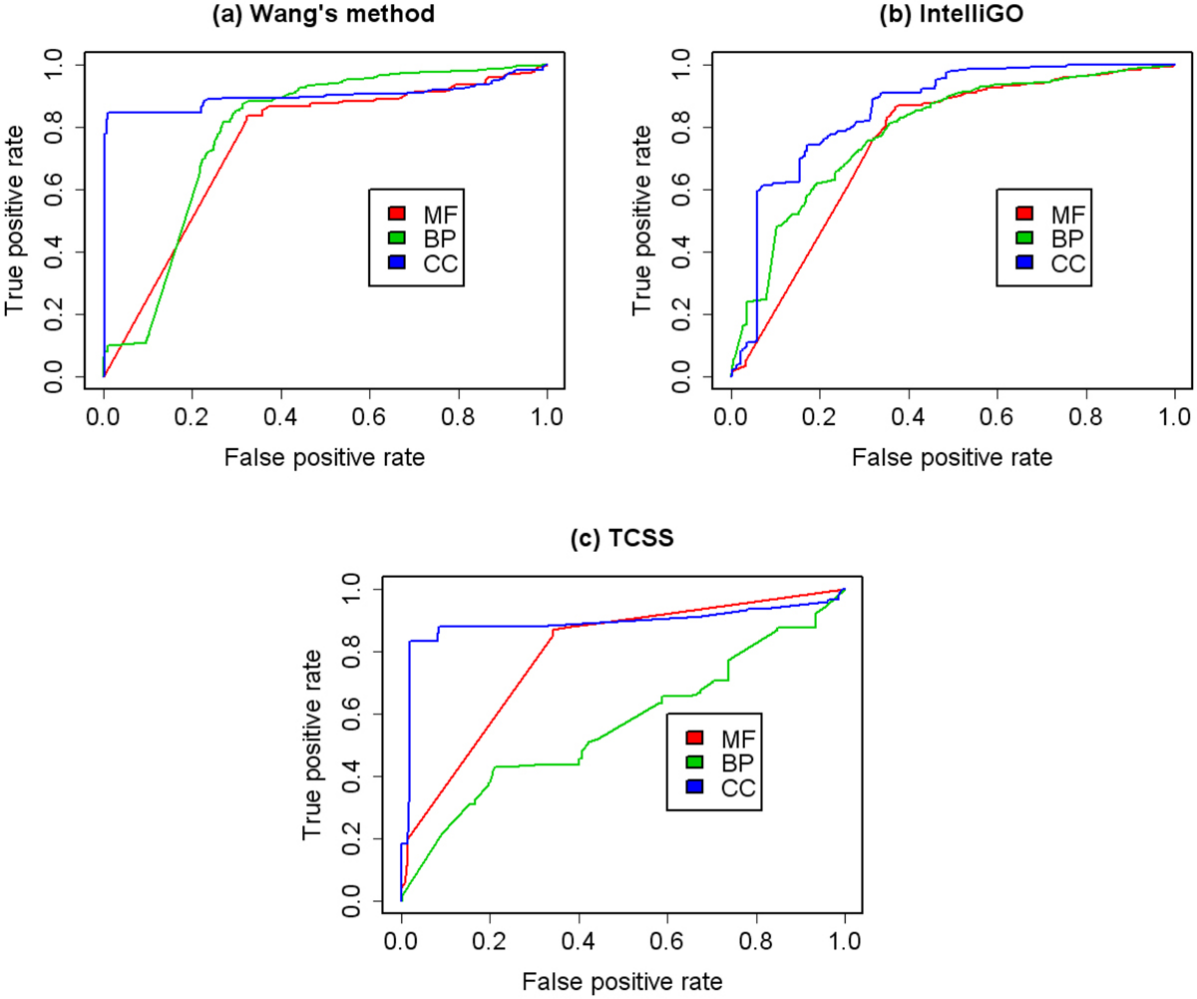
**ROC curves for co-complex data set using single GO aspect**. ROC evaluations of three GO aspects with three semantic similarity measures on the MIPS co-complex data set are shown. The evaluation was performed using only one GO aspect at a time. CC shows the overall best predictive abilities in three aspects of GO.

First, the predictive abilities of the three aspects of GO on different data sets were evaluated. We analysed the prediction performance using only one of the BP, MF and CC aspects. The receiver operating characteristic (ROC) curves are shown in Figure 2 and Figure 3. In order to assess these results quantitatively, the area under the ROC curve (AUC) of each ROC curve was calculated. The result is shown in Table 2.

**Table 2:** AUC for the yeast gold-standard PPI data sets using single GO aspect

	Binary data set			Co-complex data set		
	BP	MF	CC	BP	MF	CC
Wang	**0.9246**	0.7867	0.8696	0.7875	0.7482	**0.8994**
IntelliGO	0.8932	0.7842	0.7283	0.7882	0.7477	0.8551
TCSS	0.6178	0.6659	0.6628	0.5646	0.7891	0.8896

Tests were performed separately for biological process (BP), molecular function (MF) and cellular component (CC) ontologies in two data sets. We define similarity between two proteins as the maximum similarity found between any two GO terms that annotate them. The best ROC scores for each data set are in bold.

For the binary data set, the BP aspect shows the best performance among all three aspects in ROC analysis of three GO-based semantic similarities (Figure 2, Table 2). This result is expected. The BP aspect is related to protein interaction and thus can be used to predict them.

For the co-complex data set, the CC aspect shows the best performance in ROC analysis of three GO-based semantic similarities (Figure 3, Table 2). Since the MIPS data set is composed of protein complexes, and a protein complex can only be formed if its proteins are localized within the same compartment of the cell, terms in the CC aspect correctly reflect the functional grouping of proteins in these complexes.

**Figure 4:**
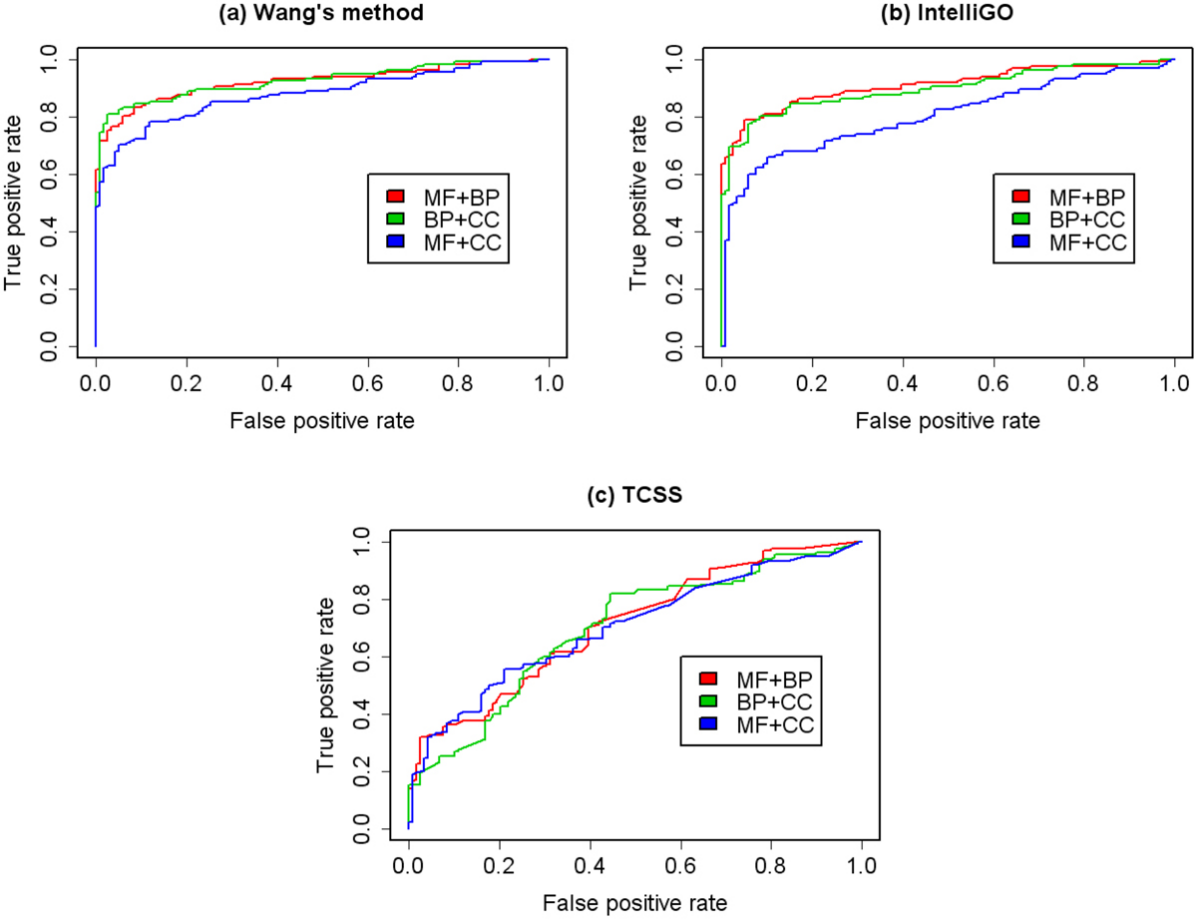
**ROC curves for binary data set using two GO aspects**. ROC evaluations of the combination of two GO aspects with three semantic similarity measures on the binary PPI data set are shown. The evaluation was performed using two of the three GO aspects at a time. In general, the prediction performance is better than that using one aspect.

**Figure 5:**
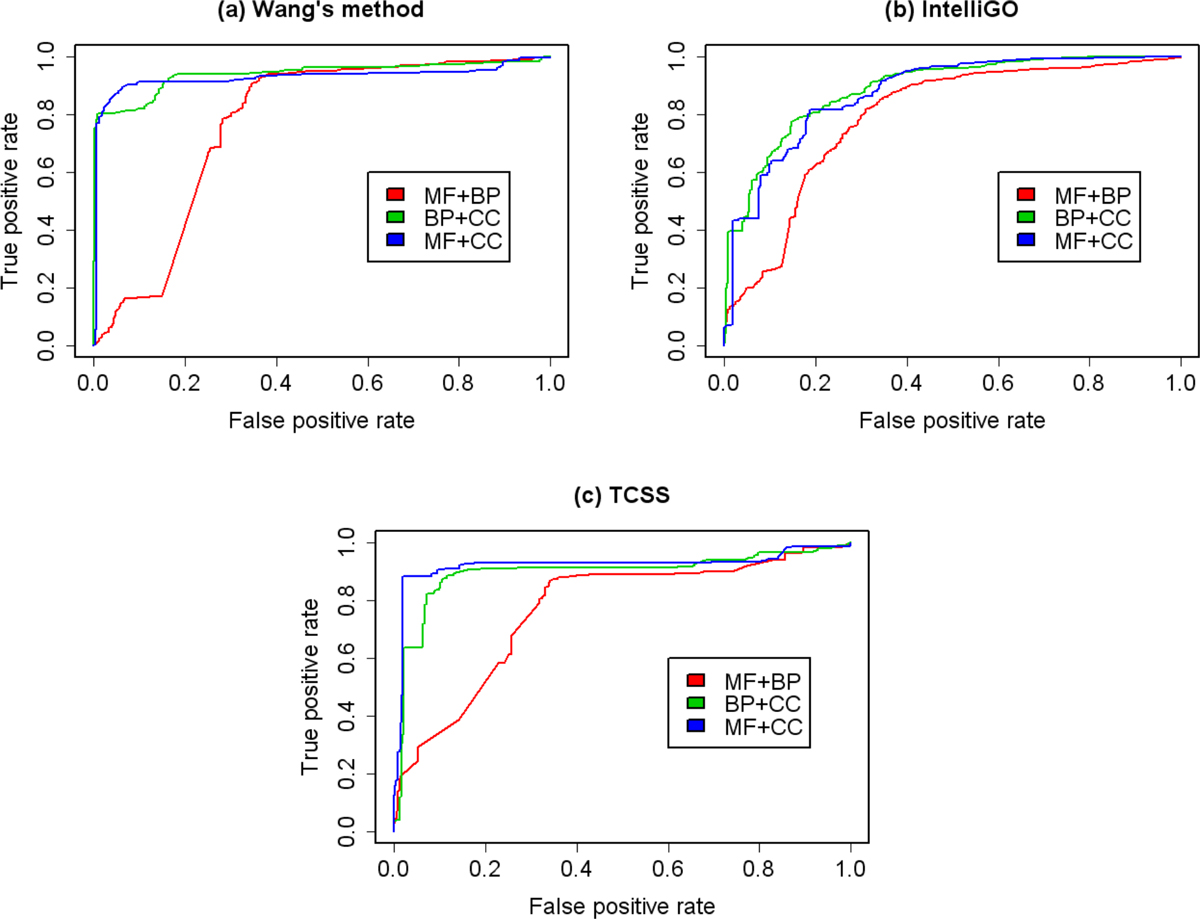
**ROC curves for co-complex data set using two GO aspects**. ROC evaluations of the combination of two GO aspects with three semantic similarity measures on the MIPS co-complex PPI data set are shown. The evaluation was performed using two of the three GO aspects at a time. In general, the prediction performance is better than that using one aspect.

**Figure 6:**
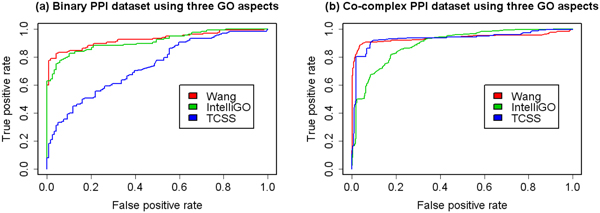
**ROC curves using three GO aspects**. ROC evaluations of the combination of all the three GO aspects with three semantic similarity measures on the binary and co-complex PPI data set are shown. In general, the prediction performance is better than that using one or two aspects.

We then analysed the prediction performance using a combination of GO aspects. The ROC curves of a combination of two aspects are shown in Figure 4 and Figure 5. The ROC curves of combination three aspects are shown in Figure 6. The AUCs of the ROC curves are shown in Table 3. The results show that by combing more than one GO aspect, our method could get a better prediction performance than using a single aspect for both binary data set and co-complex data set. And the overall best performance was achieved by combing all the three GO aspects. So it is necessary to integrate all the three GO aspects in the prediction framework.

**Table 3:** AUC for the yeast gold-standard PPI data sets using a combination of GO aspects

	Binary data set				Co-complex data set			
MF	√	√		√	√	√		√
BP	√		√	√	√		√	√
CC		√	√	√		√	√	√
Wang	0.924	0.880.	0.926	**0.927**	0.768	0.929	**0.940**	0.938
IntelliGO	0.912	0.804	0.899	0.914	0.792	0.877	0.890	0.895
TCSS	0.712	0.702	0.699	0.735	0.768	0.923	0.897	0.934

Tests were performed using a combination of three GO aspects. We define similarity between two proteins as the maximum similarity found between any two GO terms that annotate them. The best ROC scores for each data set are in bold.

### Predictive abilities of KEGG-based and topological similarities

**Figure 7:**
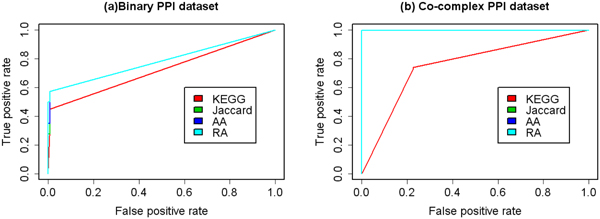
**ROC curves using KEGG-based and topological features**. ROC evaluations of the KEGG-based similarity (KEGG), Jaccard similarity (Jaccard), Adamic-Adar similarity (AA) and Resource Allocation similarity (RA) on the binary and co-complex PPI data sets are shown. The result shows that topological similarities work very well for the co-complex data set.

Then, the predictive abilities of KEGG-based similarity and three topological similarities were evaluated. For binary and co-complex data sets, the performance of KEGG-based similarity shows no big difference (Figure 7, Table 4). On the contrary, three topological similarities work perfectly for co-complex data set, but show only modest effects for binary data set. This is because the MIPS co-complex data set is composed of multi-protein complexes, and the interacting pairs are all in the same complex. The co-complex data set represents several unconnected subgraphs in the corresponding PPI network, meaning that two proteins from different complexes had no common neighbours in the PPI network. So the topological similarities of two proteins from two different complexes are zero while topological similarities of two proteins from the same complexes are not.

**Table 4:** AUC for the yeast gold-standard PPI data sets using KEGG-based and different topological similarities

	KEGG	Jaccard	AA	RA
Binary data set	0.7201	0.7819	0.7825	**0.7838**
Co-complex data set	0.7558	**0.9988**	**0.9988**	**0.9988**

Tests were performed separately for KEGG-based similarity (KEGG), Jaccard similarity (Jaccard), Adamic-Adar similarity (AA) and Resource Allocation similarity (RA) in two data sets. The best ROC scores for each data set are in bold.

### Integration of biological and topological similarities

After analysing biological and topological features separately, we integrated these heterogeneous features together.

**Figure 8:**
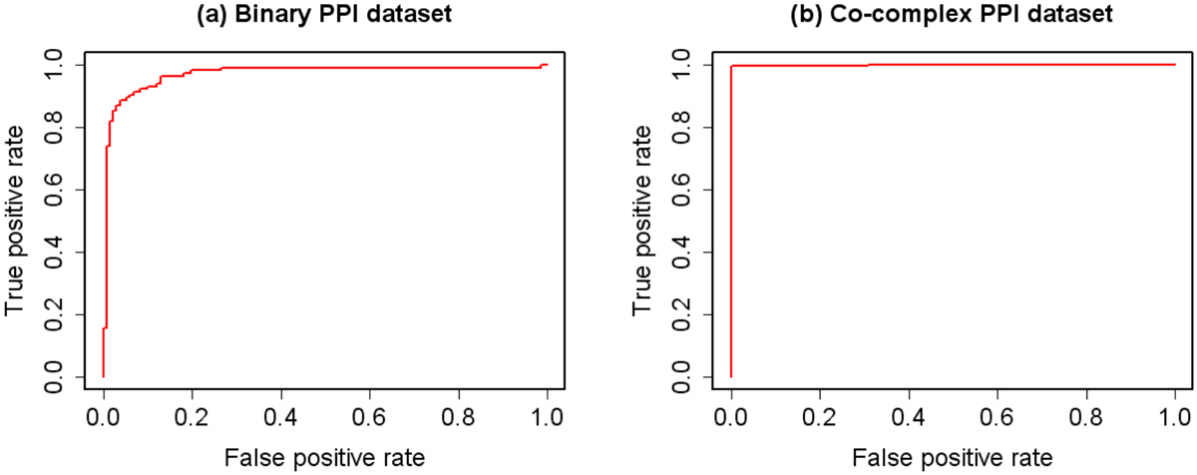
**ROC curves using a combination of GO-based, KEGG-based and topological features**. ROC evaluations of the integration of GO-based, KEGG-based and topological similarity measures on the binary and co-complex PPI data sets are shown. The result shows that integrating heterogeneous features can improve the prediction performance.

The ROC curves of two kinds of PPI data sets using GO-based, KEGG-based and topological similarities are shown in Figure 8. The AUC of binary and co-complex PPI data sets are 0.958 and 0.999.

The result shows that integrating biological and topological similarities can improve the prediction performance. So, it's necessary to integrate heterogeneous features together when dealing with the PPI prediction problem. All the features are integrated in ppiPre.

For proteins with unknown annotations in GO and KEGG, the GO-based and KEGG-based similarity measures cannot work. But the impact on these two data sets can be ignored since interactions without annotations are only 2 in the binary data set (0.19%) and 16 in MIPS data set (1.84%). However, when ppiPre is used on a large amount of proteins that are poorly annotated in GO, users should consider that the performance of ppiPre may be hampered under such situation.

### Implementation and usage

The current version of ppiPre supports 20 species. The detail of the species supported and IC data used in GO-based semantic similarities are described in [41]. The annotation data of GO and KEGG are got from the packages GO.db and KEGG.db.

ppiPre has been submitted to CRAN (Comprehensive R Archive Network) and can be installed and loaded easily in the R environment. ppiPre provides functions for calculating similarities and predicting PPIs. A summary of the functions available is shown in Table 5. Detailed descriptions and examples for all the functions are contained in the manual provided within ppiPre.

**Table 5:** Functions provided in ppiPre

Name	Description
AASim	Computes the Adamic-Adar similarity
ComputeAllEvidences	Computes biological and topological similarities
FNPre	Predict false negative interactions using topological similarities
GOKEGGSims	Computes KEGG-based similarity and GO-based similarities
IntelliGOGeneSim	Computes IntelliGO semantic similarity
JaccardSim	Computes the Jaccard similarity
KEGGSim	Computes KEGG-based similarity
RASim	Computes the Resource Allocation similarity
SVMPredict	Trains the SVM classifier, and then predict false interactions
TCSSGeneSim	Computes TCSS semantic similarity
TopologicSims	Computes all the three topological similarities
WangGeneSim	Computes Wang's semantic similarity

## Conclusions

An open-source framework ppiPre for PPI prediction is proposed in this paper. Several heterogeneous features are combined in ppiPre, including three GO-based similarities, one KEGG-based similarity and three topology-based similarities. To make the prediction, users don't need to provide additional biological data other than gold-standard PPI data.

ppiPre can be integrated into existing bioinformatics analysis pipelines in the R environment. Other features will be evaluated and integrated in future work, and the framework will be tested on PPI data of more species especially those poorly annotated in GO.

## Competing interests

The authors declare that they have no competing interests.

## Authors' contributions

LG conceived of the study, supervised the project and revised the manuscript. YD implemented the framework, performed the experiments, and drafted the manuscript. BW participated in the data analysis. All authors read and approved the final manuscript.

## Declarations

The publication of this article was funded by the NSFC (91130006, 60933009, 61202174) and the Fundamental Research Funds for the Central Universities (K5051223003, K5051223005).

This article has been published as part of *BMC Systems Biology *Volume 7 Supplement 2, 2013: Selected articles from The 6th International Conference of Computational Biology. The full contents of the supplement are available online at http://www.biomedcentral.com/bmcsystbiol/supplements/7/S2.
