# Erratum to: ppiPre: predicting protein-protein interactions by combining heterogeneous features

**DOI:** 10.1186/s12918-015-0196-5

**Published:** 2015-08-29

**Authors:** Yue Deng, Lin Gao, Bingbo Wang

**Affiliations:** School of Computer Science and Technology, Xidian University, Xi’an, 710071 PR China; Institute of Software Engineering, Xidian University, Xi’an, 710071 PR China

## Erratum

The authors wish to acknowledge that the software package associated with our Research Article [1], under the name ‘ppiPre’, re-used software code for some of its functions from an existing software package, GOSemSim [2], without proper attribution and in breach of the software’s licencing terms. Additionally we neglected to cite the article by Yu et al. [3] describing the GoSemSim software.

The software code from GoSemSim [2] is used in the implementation of two GO semantic similarity measures, TCSS and IntelliGO. ppiPre additionally implements a KEGG-based similarity measure and three topological similarity measures, and integrates features with a support vector machine.

We have now updated our software package such that it is licensed under a compatible GPL version 2 licence, and revised the package to give the appropriate attribution.

We apologize for any inconvenience this oversight may have caused.

**Availability and Requirements**

- **Project name:** ppiPre.
- **Project home page:**http://cran.r-project.org/web/packages/ppiPre/index.html.
- Operating system(s): Platform independent.
- Programming language: R.
- Other requirements: None.
- License: GPL-2.
- Any restrictions to use by non-academics: None.
